# Supplementary material for: BD-Func: a streamlined algorithm for predicting activation and inhibition of pathways
Source: PeerJ. 2013 Sep 12;1:e159. doi: 10.7717/peerj.159 (PMC3775632; doi:10.7717/peerj.159)
Supplement: Table S1 — Signatures with 80% accuracy or greater are shown in red. [file peerj-01-159-s005.doc]

**Table S1**: Accuracy of MSigDB PGR Signature

| **Cohort** | **Accuracy** | **Sensitivity** | **Specificity** | **Positive Predictive Value** | **Negative Predictive Value** |
| --- | --- | --- | --- | --- | --- |
| GSE9438  (N=31) | **96.8%** | **93.3%** | **100%** | **100%** | **94.1%** |
| Huang et al. 2003  (N=88) | 26.1% | 0% | 100% | Undefined | 26.1% |
| Chin et al. 2006  (N=117) | 43.6% | 0% | 100% | Undefined | 43.6% |
| Anders et al. 2008  (N=73) | 49.3% | 2.6% | 100% | 100% | 48.6% |
| Finak et al. 2008  (N=53) | 49.1% | 0% | 100% | Undefined | 49.5% |
| expO  (N=256) | 47.3% | 0.7% | 100% | 100% | 47.1% |
| TCGA (N=739) | 33.3% | 0% | 100% | Undefined | 33.2% |
