# Supplementary material for: BD-Func: a streamlined algorithm for predicting activation and inhibition of pathways
Source: PeerJ. 2013 Sep 12;1:e159. doi: 10.7717/peerj.159 (PMC3775632; doi:10.7717/peerj.159)
Supplement: Table S2 — Signatures with 80% accuracy or greater are shown in red. [file peerj-01-159-s006.doc]

**Table S2**: Accuracy of PGR Model Developed in This Study

| **Cohort** | **Accuracy** | **Sensitivity** | **Specificity** | **Positive Predictive Value** | **Negative Predictive Value** |
| --- | --- | --- | --- | --- | --- |
| GSE9438  (N=31) | 71.0% | 100% | 43.8% | 62.5% | 100% |
| Huang et al. 2003  (N=88) | **84.1%** | **84.6%** | **82.6%** | **93.2%** | **65.5%** |
| Chin et al. 2006  (N=117) | 75.2% | 80.3% | 68.6% | 76.8% | 72.9% |
| Anders et al. 2008  (N=73) | 75.3% | 76.3% | 74.3% | 76.3% | 74.3% |
| Finak et al. 2008  (N=53) | 71.7% | 100% | 42.3% | 64.3% | 100% |
| expO  (N=256) | **82.0%** | **78.7%** | **85.8%** | **86.3%** | **78.0%** |
| TCGA (N=739) | **85.9%** | **93.7%** | **70.3%** | **86.4%** | **84.8%** |
