# Supplementary material for: BD-Func: a streamlined algorithm for predicting activation and inhibition of pathways
Source: PeerJ. 2013 Sep 12;1:e159. doi: 10.7717/peerj.159 (PMC3775632; doi:10.7717/peerj.159)
Supplement: Table S3 — BD-Func and GSEA analysis was performed on a PC running Windows 7 (64-bit) with 24.0 GB of RAM and 2 × 5.69 GHz processors. IPA is run remotely on the vendor’s server. Run-time for IPA is for the “Core” analysis because the “Upstream Regulator” module cannot be run independently (although we would assume the run-time for this specific analysis is probably comparable to BD-Func). For IPA results, “All” means there were no filters for differential expression prior to functional enrichment, whereas “1.5×” indicates that genes needed to show a fold-change greater than 1.5 in order to be considered differentially expressed (which is the type of analysis used for the comparison between BD-Func, GSEA, and IPA – Tables 1–3). [file peerj-01-159-s007.doc]

**Table S3: Benchmark for Selected Functional Enrichment Tools**

| **Program** | **Observed Run Times** |
| --- | --- |
| **BD-Func**  **(Fold-Change)** | <1 min |
| **BD-Func**  **(Intensity)** | <1 min |
| **GSEA** | 2-25 min |
| **IPA**  **(All)** | ~20 min |
| **IPA**  **(1.5x)** | 2-4 min |
| **GATHER** | <1 min |
