# Supplementary material for: BD-Func: a streamlined algorithm for predicting activation and inhibition of pathways
Source: PeerJ. 2013 Sep 12;1:e159. doi: 10.7717/peerj.159 (PMC3775632; doi:10.7717/peerj.159)
Supplement: Table S4 [file peerj-01-159-s008.doc]

**Table S4: Size of Gene Lists used in BD-Func**

| **Signature** | **Source** | **Gene Set** | **Number of**  **Activated Genes** | **Number of**  **Inhibited Genes** |
| --- | --- | --- | --- | --- |
| TGFβ | MSigDB | Up – TGFB_UP.V1_UP  Down - TGFB_UP.V1_DN | 192 | 192 |
| mTOR | MSigDB | Up – MTOR_UP.N4.V1_UP  Down - MTOR_UP.N4.V1_DN | 196 | 193 |
| p53 | MSigDB | Up – P53_DN.V2_UP  Down - P53_DN.V2_DN | 148 | 145 |
| BRCA1 | MSigDB | Up – BRCA1_DN.V1_UP  Down - BRCA1_DN.V1_DN | 141 | 143 |
| PGR | MSigDB | Up – CLAUS_PGR_POSITIVE_MENINGIOMA_UP  Down - CLAUS_PGR_POSITIVE_MENINGIOMA_DN | 10 | 12 |
| PGR | Novel | **BD-Func Discussion Group**: COH-PGR | 421 | 179 |
| LBH589 | Novel | **BD-Func Discussion Group**: SC-LBH | 363 | 336 |
